# Coverage of a Population-Based Non-Communicable Disease Screening Program Using Lot Quality Assurance Sampling in Rural North India: A Mixed Methods Study

**DOI:** 10.7759/cureus.35330

**Published:** 2023-02-22

**Authors:** Vignesh Lognathan, Sumit Malhotra, Rakesh Kumar, Anand Krishnan, Sanjeev K Gupta, Baridalyne Nongkynrih

**Affiliations:** 1 Centre for Community Medicine, All India Institute of Medical Sciences, New Delhi, New Delhi, IND

**Keywords:** rapid epidemiological assessment methods, monitoring and evaluation, screening, non-communicable diseases, lqas

## Abstract

Aim: We aimed to estimate the coverage of a population-based Non-communicable Disease (NCD) screening program using lot quality assurance sampling (LQAS) and identify factors affecting its implementation in district Nuh of Haryana, India.

Method: A mixed-methods study was conducted with an initial LQAS coverage survey, followed by in-depth interviews. Thirty lots (villages or towns) were sampled in the district, and 20 people aged ≥ 30 years were randomly sampled from each lot. Participants were asked about receiving services under the program. Weighted coverage estimates, which is the proportion of people who had received screening services, were estimated. Using a decision value of more than nine negative responses out of 20 persons, all 30 lots were classified as good or poor performing. In-depth interviews of healthcare providers of good performing lots and district-level health officials were conducted, and factors affecting program implementation were identified.

Findings: Six hundred participants were interviewed (mean age of 44.8 years, 57.2% women). The proportion of people who reported having undergone screening for diabetes or hypertension was 2.1%, and all lots performed poorly based on decision value. Key factors affecting the program were leadership, prioritization of NCD activities, ensuring human resource and material requirements, regular incentives, qualities of workers, and community engagement.

Conclusion: The screening coverage under the population-based NCD screening program was low in district Nuh, Haryana. This needs to be improved by addressing the identified health system and community-related factors.

## Introduction

The Population-Based Screening (PBS) program was rolled out through the National Program for the Prevention and Control of Cancer, Diabetes, Cardiovascular Diseases, and Stroke (NPCDCS) in 2017 to integrate health promotion, screening, and management of five common non-communicable diseases (NCDs): diabetes, hypertension, oral cancer, breast cancer, and cervical cancer, for people who are ≥ 30 years of age [1]. This program is integrated with the latest Health & Wellness Centres (HWCs) initiative as part of the 12 expanded range of services to provide Comprehensive Primary Health Care (CPHC). As per the health ministry's annual report (2019-20), the PBS program has been implemented in 219 of the 742 districts in India [2]. As of 2020, more than 80% of Medical Officers (MO), Auxiliary Nurse Midwife (ANM), Staff nurses (SN), and Accredited Social Health Activists (ASHA) in Haryana have undergone training for implementing PBS [3]. Nuh (previously Mewat) is a high-priority district (commonly known as an aspirational district) in Haryana State with a population of 10,89,406, which is predominantly rural (90%) [4,5]. Nuh is prioritized for the operationalization of HWCs and has the highest number of functional HWCs in Haryana [3]. In the district Nuh, the NPCDCS-PBS program was implemented in 2018.

The targets for achieving coverage for screening under the program are 50% in year one, 65% in year two, and 80% in year three post-implementation [1]. It is desirable to design coverage surveys that utilize existing resources and can give rapid results to aid local health officials in making implementation decisions. Lot Quality Assurance Sampling (LQAS) is one such type of Rapid Epidemiological Assessment (REA) method, where the population under survey is divided into non-overlapping subpopulations (lots) for sampling and surveyed for coverage of a program [6]. It has been used to estimate immunization coverage, evaluate communicable disease control status, assess the performance of community health workers, etc. [7,8]. This method can provide coverage estimates for the whole population under survey and for each such sub-population or lot to redirect attention to where it is needed [9,10]. Being a rapid evaluation tool and amenable to use by health staff, it can be adapted for periodic coverage monitoring of the NCD screening program [11].

To estimate the coverage of population enumeration and the Community Based Assessment Checklist (CBAC) by ASHA under the NPCDCS PBS program in Nuh (Mewat) district, Haryana, using the LQAS technique, and to determine the factors facilitating and limiting implementation and coverage of the program.

## Materials and methods

Study characteristics

A mixed methods study consisting of two phases was designed to meet the objectives: an initial quantitative phase to estimate coverage and a qualitative phase where in-depth interviews were conducted to explain and supplement the quantitative survey findings. Lot quality assurance sampling (LQAS) was used to perform the coverage estimation of population enumeration, administration of CBAC, and screening activities by ASHA and ANM in the villages and towns of district Nuh, Haryana, India. The study was performed during the period December 2019 and January 2020. The study population was people who were ≥ 30 years of age and residents of the village/town for > six months.

Sample size and sampling

For the desired level of confidence and accuracy for the coverage estimate of 4% and 95%, respectively, World Health Organization (WHO) manual for using LQAS was used to estimate a sample size of 600 [10]. A lot was defined as a village or town in the district. Out of the 443 villages and four towns in Nuh as per Census 2011, 27 villages and three towns were selected by probability proportional to size, giving a total of 30 lots (20 people to be sampled in each lot) [5]. The map-grid method was used in each lot to select 20 sampling areas as sampling frames or household lists were unavailable [10]. Each sampling area was approached to its approximate center using Geographical Positioning System (GPS); roads were counted north → east → south → west, and one was randomly selected. If the sampling area didn’t have a house, another sampling area was selected. In the selected road, a total number of houses were counted, and one house was randomly selected. In the selected house, a total number of eligible participants was counted, and one was randomly selected and approached to participate in the study. In case the selected house was locked or did not have any eligible person, or when the selected person was not available after two visits or didn’t consent to the study, an immediately adjacent house in the forward direction was approached. Separate random tables were used for sampling at different levels. These selected participants were asked whether they had received services under the program, using cues and specifiers to elicit the status of enumeration & CBAC administration by ASHA and the status of screening by ANM. A pretested semi-structured interview schedule in Hindi and Epicollect5 application were used for data collection.

Quantitative analysis

The proportion for categorical variables and mean with standard deviation for continuous variables were calculated for descriptive analyses. Sample weight was calculated as the inverse of the probability of selection (the product of the probability of selecting a lot and the probability of choosing an individual in a lot) and applied to all the individuals in the dataset before analysis. Coverage estimates with a 95% confidence interval for various indicators were calculated using the command for estimating proportions for survey data in StataCorp. 2011. Stata Statistical Software: Release 12. College Station, TX: StataCorp LP. In addition to evaluating the overall coverage, lots were classified as good and poor performing for various indicators using a decision value of nine, obtained from Lemeshow & Taber LQAS tables [10]. A decision value of nine meant that when more than nine participants in a lot gave a negative response for an indicator, the lot was classified as poor performing.

Qualitative methods

The qualitative part was designed to supplement and explain the survey's quantitative findings and determine the factors facilitating and limiting the program. In-depth interviews were conducted over the telephone or in person using interview guides from July to September 2020. One of the good-performing lots was selected for elucidating the facilitating factors. As the investigator couldn’t contact the health workers in poor-performing lots due to COVID-19 lockdown restrictions, interviews with district health officials were used to identify barriers to program implementation. A total of three interviews were conducted, one each for an ASHA, a CHO, and a MO from one of the good-performing lots. And two interviews were conducted with one district NCD Officer and the district's Chief Medical Officer (CMO). The persons in charge during the first year of program implementation, were contacted and interviews were arranged.

Qualitative analysis

Interviews were conducted in Hindi and English. Interview audio records were transcribed into Hindi and translated into English using Microsoft Word (Redmond, USA). Scripts were coded manually by the deductive coding method. Facilitators and barriers to program implementation and coverage were then identified. Themes were formed by grouping the codes to identify critical areas of focus for the overall improvement of program implementation based on the framework of Health System Building Blocks and the role of the community.

Ethics approval

Ethical approval for conducting the study was obtained from the Institute Ethics Committee, AIIMS New Delhi (Number: IECPG-522/14.11.2018, OT-1/29.08.2019). Informed consent was obtained from the village or town representatives and study participants.

## Results

LQAS survey results

A total of 622 houses were approached for the study. Four houses were locked, and five had no eligible persons for the study. Of the 613 houses with at least one eligible person, four houses did not have the selected participants available for the interview even after two visits. Nine people did not consent to participate in the study. Finally, a total of 600 participants were interviewed for the survey. The mean ± standard deviation of the age of the participants was 44.8 ± 12.2 years. Most of the participants were women (57.2%), housewives (55.2%), followed by laborers (15.8%), had no schooling (68.5%), and belonged to the Muslim religion (80.2%). According to the Udai Pareek scale [12], most families were classified as lower class (55.7%), followed by the lower middle class (44%). Of the 600 participants, 12 (2.0%) reported having diabetes mellitus, 22 (3.7%) reported having hypertension, and three (0.5%) reported having both diabetes and hypertension. Of the 12 diabetes and 22 hypertensives, eight and 12 said they were currently taking medications for these conditions.

Coverage of population-based screening program activities

The coverage of the key activities under the population-based screening program is shown in Table 1. The proportion of people for whom ASHA had asked details related to population enumeration, i.e., line listing of family members and their identity details, fuel, and water usage, was 66.5% (95% CI: 60.9-72.0), with 25 of the 30 lots performing acceptably on this indicator. The estimated proportion of people who self-reported to have undergone screening for diabetes and hypertension was only 2.1% (95% CI: 0.01-4.3), and all 30 lots performed poorly for the same.

**Table 1:** Coverage of population-based screening program activities ASHA: Accredited Social Health Activist, ANM: Auxillary Nurse Midwife, CBAC: Community Based Assessment Checklist

No.	Indicator	Estimated overall coverage among eligible persons in Nuh district, % (95% CI)	Good performing lots (> 10/20 positive response/lot)	Poor performing lots (< 11/20 positive response/lot)
1	Recognizing ASHA(s) in their village	89.2 (85.6–92.7)	30/30	None
2	ASHA asked population enumeration related details	66.5 (60.9–72.0)	25/30	5/30
3	ASHA asked disease status details	8.9 (5.8–12.0)	None	30/30
4	ASHA asked details related to CBA checklist	2.5 (1.0–4.0)	None	30/30
5	ASHA measured waist circumference	0.9 (0–1.8)	None	30/30
6	ASHA informed the need for screening for Diabetes & Hypertension	4.5 (0.1–8.9)	None	30/30
7	ASHA provided information about a screening camp	5.3 (0–11.3)	1/30	29/30
8	Screened for diabetes and/or hypertension in the camp conducted through ASHA and/or ANM	2.1 (0.01–4.3)	None	30/30

Of the 12 participants out of 600 who reported having undergone screening for diabetes or hypertension, two participants were detected to be screen positive (one person with diabetes and another hypertensive). They were referred to Primary Health Centre (PHC) for diagnosis and were then diagnosed with the disease.

Results from in-depth interviews

**Figure 1:**
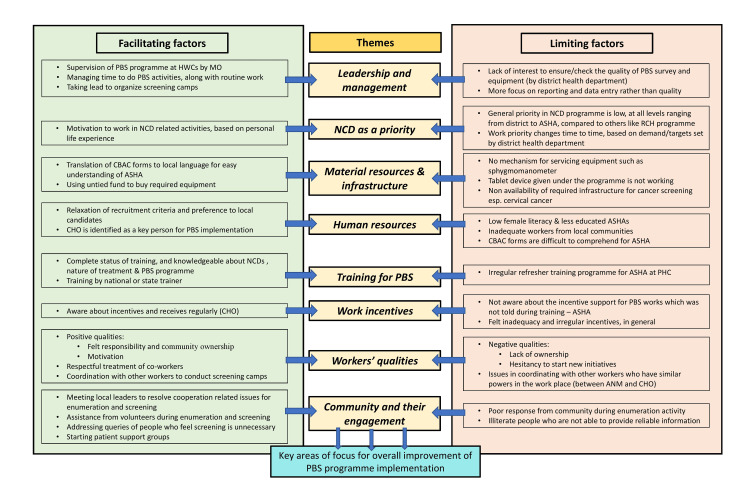
Framework showing the factors which facilitate and limit the implementation of the population-based screening program HWCs: Health & Wellness Centers, MO: Medical Officer, PBS: Population-Based Screening, NCD: Non-Communicable Diseases, CBAC: Community-Based Assessment Checklist, ASHA: Accredited Social Health Activist, CHO: Community Health Officer, RCH: Reproductive and Child Health, PHC: Primary Health Centre, ANM: Auxiliary Nurse and Midwife

A total of five in-depth interviews were conducted. On coding the scripts, facilitating and limiting factors for program implementation and coverage were identified. These factors, whichever recurred across the interviews, were grouped to identify key areas of focus for overall improvement of program implementation, giving eight themes. The various facilitating factors, limiting factors and themes identified are shown in Figure 1.

Key facilitating factors that emerged were i) Leadership at all levels of implementation to conduct the enumeration activity or organize the screening camps, and work management skills, ii) Prioritization of NCD activities, iii) Arrangements to ensure human resource and material requirements, iv) Identify CHO as a critical person coordinating the PBS activities, v) Regular incentives for various PBS-related activities to CHO, vi) Positive worker qualities and coordination capabilities, and vii) Engaging community and beneficiaries by informing them of the need and resolving their queries. Key limiting factors for the program implementation were i) Low priority is given to NCD activity relative to other programs, at different levels, from district to ASHA, ii) Lack of adequate equipment and infrastructure to perform screening activities, iii) An inadequate number of health workers from the local community in the cadre of ANM and CHO, iv) Nonpayment of incentives for PBS activities to ASHAs and their need for awareness about the same, v) Lack of ownership over their community, hesitancy to start new initiatives, and vi) Poor response from the community. In addition to these factors, it is imperative to note that lockdown measures implemented for COVID-19 pandemic control efforts led to a temporary cessation of all screening-related activities in the village.

Eight themes were identified at the end of the thematic analysis: leadership and management, NCD as a priority, material resources and infrastructure, human resources, training for PBS, work incentives, workers’ qualities, community characteristics, and community engagement. These themes could be considered critical areas of focus for improving the implementation and coverage of the PBS program when a program review is performed at the district and state levels.

## Discussion

Our LQAS survey found that the estimated coverage of screening under the population-based NCD screening program was less than the desired target of 50% in district Nuh, Haryana. Using the LQAS technique permitted the classification of villages or towns into good or poor performance for the various program activities and explored multiple factors that could be attributable to their performance. Designing an LQAS survey has made the study operationalizable in a setting without a prior sampling frame, which otherwise would have been difficult through a survey using usual sampling methods. Also, it was found that this technique gives results rapidly, making it a preferable method choice. Studies that assess the feasibility and efficiency of using LQAS as part of routine monitoring systems at the district level could give more evidence about its overall utility.

A similar study by Nambiar D et al. assessed the coverage of screening for diabetes and hypertension under the PBS program in four districts of two states using the LQAS technique [13]. However, a lot was defined as the area served by one ANM. It was found that the coverage levels of blood pressure screening were less than the desired threshold in both Delhi (58.9% in Central and 61.5% in South, both less than 65%) and Uttar Pradesh (30.9% in Shrawasti and 33.4% in Jhansi, both less than 50%). Blood sugar screening coverage levels were much less than the desired threshold in Delhi (47.4% in Central and 53.1% in South, both less than 65%) and Uttar Pradesh (13.7% in Shrawasti and 20.4% in Jhansi, both less than 50%) [13]. Although the coverage of screening services was lower than the desired target in both Nambiar et al. and this study, the magnitude of the difference between theirs and this study is significant [13]. This could be attributed to differences in implementation period and health system or community-related factors between states and districts. A health system readiness assessment for rolling out of the universal screening, prevention, and management of common NCDs was done by the National Health Systems Research Centre (NHSRC) reported that there was a low level of readiness, inadequate/no training for ANMs and MOs (during early 2018), screening activities not yet rolled out and cultural barriers for rolling out the program [14]. Although this assessment was conducted a few months earlier to our study, their results are comparable to the findings from the qualitative results of our research. There was no quantitative coverage estimation for various programmatic areas such as population enumeration, CBAC assessment, or screening.

The study's strengths were that random sampling methods were used at all sampling levels (village or town, sampling area, road, house, eligible participant), increasing the coverage estimate's generalizability. Also, the random selection at the village level to select sampling areas gave an equal probability of communities with different socio-demographic statuses being chosen for the study. The qualitative method through in-depth interviews helped to supplement the quantitative findings.

The study's limitations include that during the data collection period, there was social unrest in the community, which could have affected the validity of the findings [15]. Also, in-depth interviews couldn’t be conducted in the poor-performing lots given the COVID-19 pandemic, which might have affected data saturation.

## Conclusions

The screening coverage under the population-based NCD screening program was low in district Nuh, Haryana. This needs to be improved by addressing the identified health system and community-related factors. Making NCD a priority, leadership and management, provision of material and human resources, training for PBS, work incentives, workers’ qualities, and community engagement could be key areas of focus for improving the implementation and coverage of a community-based NCD screening program in India.
